# DWI Combined With Hepatobiliary-Phase Enhanced Imaging Can Better Differentiate Cholangiocarcinoma From Atypical Liver Abscesses

**DOI:** 10.3389/fonc.2022.723089

**Published:** 2022-05-13

**Authors:** Li-Hong Xing, Li-Yong Zhuo, Yu Zhang, Xi Ma, Ze-Peng Ma, Ying-Jia Zhao, Xiao-Ping Yin, Bu-Lang Gao

**Affiliations:** ^1^ Department of CT/MRI Room, Affiliated Hospital of Hebei University, and Key Laboratory of Cancer Radiotherapy and Chemotherapy Mechanism and Regulations, Baoding, China; ^2^ School of Clinical Medicine of Hebei University, Baoding, China

**Keywords:** intrahepatic mass-forming cholangiocarcinoma, atypical liver abscess, DWI, hepatobiliary phase, enhanced imaging

## Abstract

**Objective:**

To investigate the value of diffusion-weighted imaging (DWI) combined with the hepatobiliary phase (HBP) Gd-BOPTA enhancement in differentiating intrahepatic mass-forming cholangiocarcinoma (IMCC) from atypical liver abscess.

**Materials and Methods:**

A retrospective analysis was performed on 43 patients with IMCCs (IMCC group) and 25 patients with atypical liver abscesses (liver abscess group). The DWI signal, the absolute value of the contrast noise ratio (│CNR│) at the HBP, and visibility were analyzed.

**Results:**

A relatively high DWI signal and a relatively high peripheral signal were presented in 29 patients (67.5%) in the IMCC group, and a relatively high DWI signal was displayed in 15 patients (60.0%) in the atypical abscess group with a relatively high peripheral signal in only one (6.7%) patient and a relatively high central signal in 14 (93.3%, 14/15). A significant (P<0.001) difference existed in the pattern of signal between the two groups of patients. On T2WI, IMCC was mainly manifested by homogeneous signal (53.5%), whereas atypical liver abscesses were mainly manifested by heterogeneous signal and relatively high central signal (32%, and 64%), with a significant difference (*P*<0.001) in T2WI imaging presentation between the two groups. On the HBP imaging, there was a statistically significant difference in peripheral │CNR│ (*P*< 0.001) and visibility between two groups. The sensitivity of the HBP imaging was significantly (*P*=0.002) higher than that of DWI. The sensitivity and accuracy of DWI combined with enhanced HBP imaging were significantly (*P*=0.002 and *P*<0.001) higher than those of either HBP imaging or DWI alone.

**Conclusion:**

Intrahepatic mass-forming cholangiocarcinoma and atypical liver abscesses exhibit different imaging signals, and combination of DWI and hepatobiliary-phase enhanced imaging has higher sensitivity and accuracy than either technique in differentiating intrahepatic mass-forming cholangiocarcinoma from atypical liver abscesses.

## Introduction

Biliary tract cancer (BTC) encompasses a group of rare, heterogeneous, and highly aggressive hepatobiliary malignancies, including ampulla of Vater cancer, gallbladder cancer, extrahepatic cholangiocarcinoma, and intrahepatic cholangiocarcinoma (1). A significant proportion of patients with BTC were found to be unresectable during exploratory laparotomy, and potentially curative surgical resection is possible only in approximately 25% of these patients, for which the recurrence rates are extremely high even after radical surgical resection (2). Intrahepatic mass-forming cholangiocarcinoma (IMCC) is the most common subtype of intrahepatic cholangiocarcinoma. Liver abscesses with a central area of necrotic liquefaction, a peripheral “ring-target sign”, and focal intrahepatic gas on computed tomography (CT) images are referred to as the typical liver abscess (3, 4), whereas others without these typical imaging features are atypical ones (5). IMCC may present with fever as the first clinical symptom, similar to that of liver abscesses, whereas atypical liver abscesses may have no clinical manifestations or symptoms, similar to those of a liver tumor (3). Because both IMCCs and atypical liver abscesses may have the following characteristics of a single lesion, edge ring enhancement, and continuous enhancement on conventional CT imaging, it is very difficult to distinguish these two disease entities (5). High signal on T2WI, enhancement degree of edge parenchyma at the arterial phase, enhancement speed of central parenchyma at a late enhancement phase, and dynamic enhancement patterns on conventional magnetic resonance imaging (MRI) may have some values in differentiation of the two diseases (5–7). However, the two diseases have varied imaging morphology, density, signal, enhancement mode, and prognoses (8), necessitating different treatments.

IMCCs may exhibit a high signal on MRI diffusion-weighted imaging (DWI), whereas atypical liver abscesses have a high signal in the cystic region (9). In areas around the IMCC lesions, tumor tissue is rich with limited diffusion of water molecules, whereas in the central region, tumor cells are sparse and are primarily fibrostromal and fibroblast cells (9, 10). IMCCs may present with an overall high signal on DWI (6), but when the tumor lesion is simultaneously infected with bacteria, the lesion may display heterogeneous signals which can be easily confused with atypical liver abscesses (11). On the contrary, an atypical liver abscess with less or no necrosis, especially at the early stage of development, may present with a high signal or heterogeneous signal, similar to that of a tumor (11, 12).

Application of a hepatocyte specific contrast agent, such as Gadobenate dimeglumine (Gd-BOPTA), has a certain value in the diagnosis of liver lesions. Low signal at the hepatobiliary phase (HBP) is a comparative characteristic and an independent sign of malignant liver lesions (13). At the HBP, IMCCs may demonstrate a low peripheral signal and a high central signal, which was referred to as the “target sign” (9, 14–18), whereas the peripheral part of an atypical liver abscess exhibits continuous enhancement (8). However, the target ring sign may not always be demonstrated on the image of IMCCs, and in cases of cirrhosis or abnormal liver function, IMCCs may have a low signal (19).

DWI and imaging at the HBP may thus have some values in differentiating IMCCs from atypical liver abscesses, but the correct diagnosis may be missed for either single imaging method. It was thus speculated that the combination of the two would be effective in differentiating the two diseases. This study was consequently performed to investigate the value of DWI combined with the imaging at the HBP of Gd-BOPTA enhancement for the diagnosis of IMCCs and atypical liver abscesses.

## Materials and Methods

### Subjects

**Figure 1:**
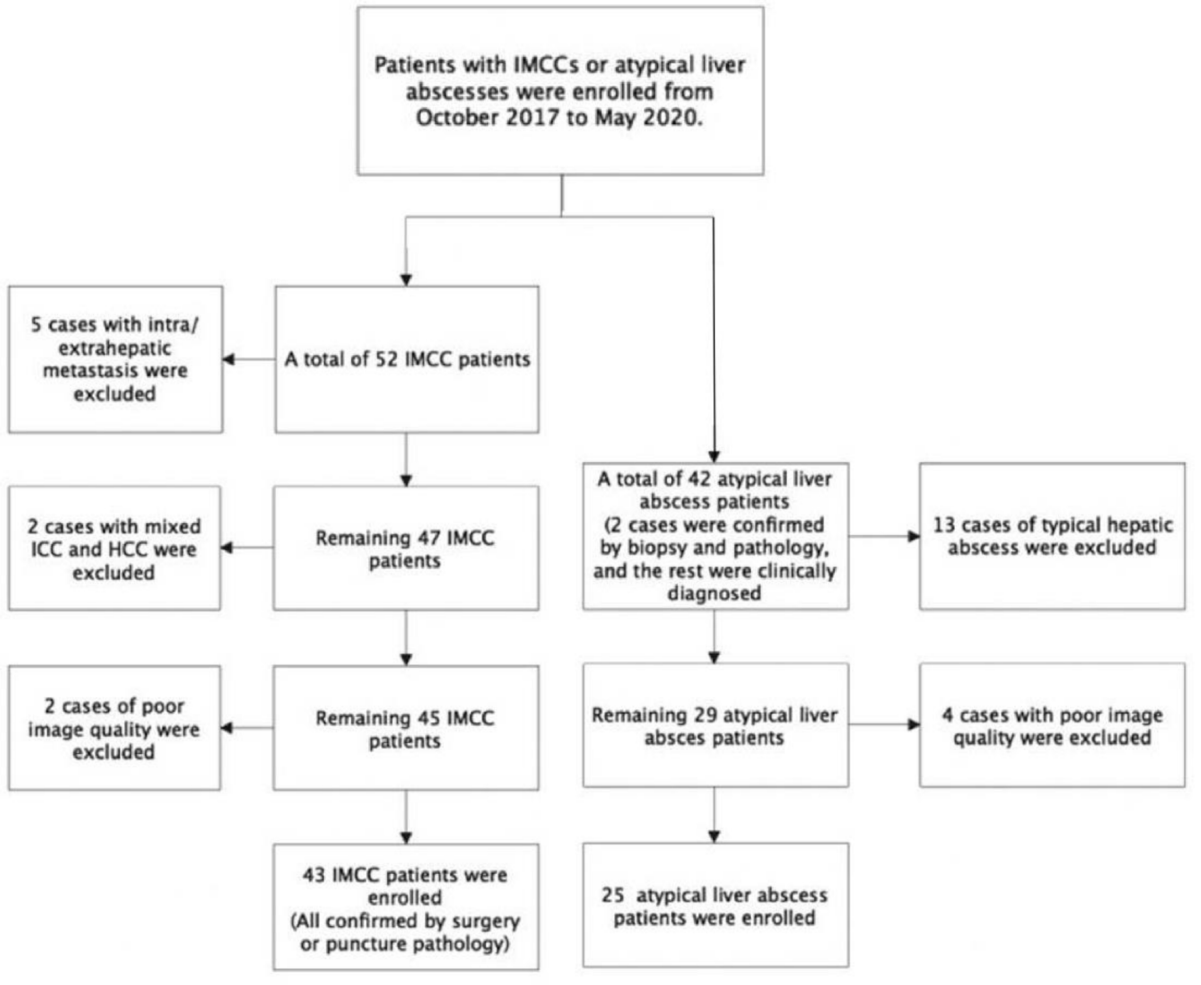
Process of patient enrollment in different groups.

This retrospective cohort study was approved by the ethics committee of our hospital (201710281), and all patients had provided signed informed consent to participate. Patients with IMCCs or atypical liver abscesses were enrolled from October 2017 to May 2020. For the IMCC group, the inclusion criteria were patients with IMCCs confirmed by surgery or percutaneous biopsy and with complete pre-operative liver contrast enhanced MRI. The exclusion criteria were patients with liver metastasis, coexistence of hepatocellular carcinoma confirmed by pathology, and poor image quality precluding analysis. Forty-three patients with IMCCs were enrolled including 25 males and 18 females with a mean age 63 ± 10 years (**Figure 1**). For the atypical liver abscess group, the inclusion criteria were patients with liver abscesses confirmed by reduced lesion volume on reexamination imaging after surgery, percutaneous biopsy or treatment, and patients who had undergone MRI examination before pathology. The exclusion criteria were patients with typical liver abscesses (3, 4) and poor image quality precluding analysis. Twenty-five patients with atypical liver abscesses were enrolled including 12 males and 13 females with a mean age 59 ± 11 years (**Figure 1**). Among the 25 cases with atypical liver abscesses, two were confirmed as inflammatory lesions by biopsy pathology, whereas the remaining 23 patients had lesions which shrank or disappeared on ultrasound or CT follow-up imaging.

### MRI Examination

**Figure 2:**
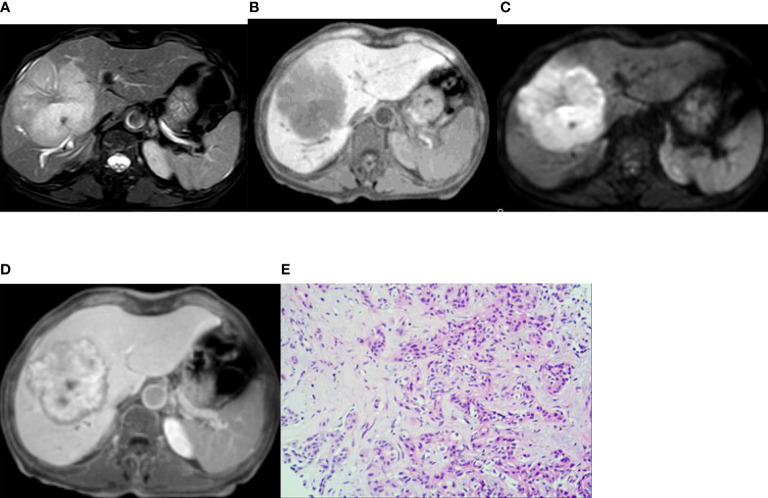
Imaging of intrahepatic mass-forming cholangiocarcinoma. A 66-year-old woman had cholangiocarcinoma with fatigue, no obvious cause of poor appetite, and laboratory examination of CA19-9 13455 U/mL. **(A, B)** (T2WI and T1WI) showed a lobulated mass with a long T1 and slightly longer T2 signal with clear boundaries. **(C)** The lesion showed a peripheral relatively high signal on DWI image. **(D)** On the hepatobiliary-phase enhanced imaging, the peripheral signal was low, whereas the central signal was high. The │CNR│ was 34.92 in the peripheral region but 20.94 in the central. The visibility score was 5. **(E)** Pathological sections showed heterogeneous epithelial cells in the fibrous tissues, some of which were glandular and in cords, with mucinous degeneration in the interstitial fibrous tissues. The pathologic diagnosis was cholangiocarcinoma (medium–poorly differentiated).

**Figure 3:**
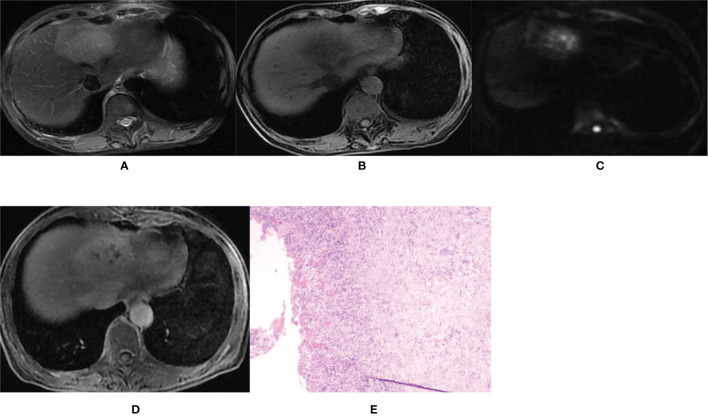
Imaging of hepatic abscess. A 60-year-old female patient was admitted to our hospital mainly due to a fever for 4 days with the white blood cell count of 9.38×10^9^/L and the percentage of neutrophils of 83.1%. **(A, B)** T2WI and T1WI showed a round mass shadow in the left lobe with a clear boundary, slightly longer T1 and slightly longer T2 signal. **(C)** The DWI image showed multiple high spotted signals in the center, and the central signal was higher than the peripheral signal. **(D)** On the hepatobiliary-phase enhanced imaging, the lesion showed a slightly high peripheral signal and patchy low signal in the center. The │CNR│ was 17.17 in the peripheral region but 13.21 in the central. The visibility score was 3. **(E)** Pathological sections showed fibrocystic wall tissue with acute and chronic inflammatory cell infiltration, but no clear lining epithelium. Some small vessels were dilated and congested, which was consistent with the wall of a liver abscess. The pathologic diagnosis was a hepatic abscess.

GE Discovery MR 750 3.0T scanning equipment (GE Medical Systems, Milwaukee, WI, USA) and Philips Achieva 1.5T MR scanning equipment (Amsterdam, The Netherlands) were used. Axial fat suppression T2WI (**Figures 2A**, **3A**), in-phase T1WI and out-phase T1WI (**Figures 2B**, **3B**), axial DWI (**Figures 2C**, **3C**), axial VIBE mask (pre-scan T1WI), axial VIBE phase III dynamic enhanced scan, and HBP scan (**Figures 2D**, **3D**) were performed. The scanning parameters for GE Discovery MR 750 3.0T scanner were: (1) T2WI: TR 8000ms, TE 68.90ms, NEX 2, layer thickness 5.0mm, layer spacing 6mm, matrix 320×320, and FOV 40cm×40cm; (2) T1WI: TR 3.70ms, TE 2.23ms, NEX 0.70, layer thickness 5.0mm, layer spacing 2.5mm, matrix 260×224, and FOV 40cm×40cm; (3) DWI: TR 7500 ms, TE 80 ms, NEX 6, layer thickness 5.0mm, layer spacing 6mm, matrix 128×130, and FOV 40cm×40cm; (4) VIBE scanning: TR 3.70ms, TE 1.67ms, NEX 0.70, layer thickness 5.0mm, layer spacing 2.5mm, matrix 260×224, and FOV 40cm×40cm. The scanning parameters for Philips Achieva 1.5T MR scanner were: (1) T2WI: TR 334ms, TE 65ms, NEX 2, layer thickness 5.0mm, layer spacing 6mm, matrix 220×252, and FOV 40.2cm×40.2cm; (2) T1WI: TR 175 ms, TE 4 ms, NEX 1, layer thickness 7.0 mm, layer spacing 8.0mm, matrix 260×202, and FOV 37.5cm×37.5cm; (3) DWI: TR 1574 ms, TE 64 ms, NEX 6, layer thickness 7.0 mm, layer spacing 8.0 mm, matrix 124×124, and FOV 37.5cm×37.5 cm; (4) VIBE scanning: TR 3.96 ms, TE 1.87 ms, NEX 1, layer thickness 7.0 mm, layer spacing 8.0mm, matrix 192×192, and FOV 34.7cm×34.7cm.

Gd-BOPTA was used as the contrast agent and injected through the cubital vein, with a dose of 0.1 mmol/kg and an injection flow rate of 2ml/s, followed by a wash with at least 20ml physiological saline at the same rate. Early and late arterial stages and portal and equilibrium phase scans were performed at 15–20s, 35–40s, 60 s and 180 s, respectively, after contrast injection. A 90-minute delay was required for the hepatobiliary scan.

### Image Analysis

Images were reviewed by two imaging diagnostic physicians with ten years of experience without knowledge of the pathological results. In the case of disagreement, consensus was reached through consultation. The MRI findings were described according to the following characteristic parameters. (1) General conditions: morphology regularity, boundary clarity, capsule existence, necrotic cystic degeneration, steatosis, and existence of bleeding. (2)DWI signal pattern: homogeneous or heterogeneous high signal, relatively high central or peripheral signal (compared with the central signal, the peripheral higher signal is defined as the relatively high peripheral signal; compared with the peripheral signal, the central higher signal is defined as the relatively high central signal). (3)T2WI signal pattern: homogeneous or heterogeneous high signal, relatively high central or peripheral signal. (4) HBP signal pattern: homogeneous or heterogeneous high signal, homogeneous low signal, high or low central or peripheral signal. Lesions’ signal intensity (SI_lesion_), normal liver parenchyma performance (SI_liver_), and background signal intensity (SI_background_) were measured. The measurement methods were as follows: first, the lesions suspected to have bleeding were removed from the mask. The region of interest (ROI) was placed both peripherally and centrally. The signal of surrounding normal liver parenchyma was measured on the same liver lobe at the maximal level of the lesion, within a radius of 40mm from the lesion. Hepatic vessels and biliary tract were avoided as much as possible. All data were measured three times and averaged. The SI_background_ was measured in the right anterior area of the abdominal wall with the standard deviation of SI_background_ set as the calculated value (SD.SI_background_). According to the data obtained, the absolute value of the contrast to noise ratio (CNR) for hepatobiliary lesions was calculated from the formula: │CNR│ = │SI_lesion_ – SI_liver_│/SD.SI_background_× 100%. (5) Assessment of visibility of hepatobiliary lesions was performed based on how clearly the edges and contours of the lesion appeared on the MRI image, with scores on a 5-point scale: 1 = completely invisible, 2 = very obscure or hard to see, 3 = fair, 4 = good display, and 5 = very good.

### Statistical Methods

The SPSS 25.0 statistical software (IBM, Chicago, IL, USA) was used. Measurement data were presented as mean ± standard deviation (SD). The independent sample t test was used to compare the measurement data between groups. Classified data were presented as numbers and frequency and analyzed with the Chi square test or Fisher’s exact probability method. The P values of multigroup Chi square were corrected by Bonferroni. The significant P value was set as <0.05.

## Results

There were no statistically significant (P>0.05) differences in gender, age, lesion location, maximal diameter, morphology, and boundary between patients with IMCCs and liver abscesses, however, a significant (*P*<0.05) difference existed in the cystic degeneration characteristic (**Table 1**).

**Table 1:** Demography and clinical information.

		group		*t l* *χ* ^2^	*P*
		IMCC	atypical liver abscess		
sex	male	25	12	0.801	0.426
	female	18	13		
age (years, mean ± SD)		63±10	59±11	1.569	0.121
location	left	13	6	0.704	0.703
	right	18	13		
	left and right	12	6		
largest diameter (cm,mean ± SD)		5.96±2.9	5.98±3.2	-0.023	0.981
morphology	regular	28	21	2.800	0.094
	irregular	15	4		
boundary	clear	38	18	2.916	0.088
	fuzzy	5	7		
necrotic or cystic degeneration	yes	0	20	–	0.002
	no	43	5		

The statistical method was the Fisher exact probability method, without specific test value. IMCC, intrahepatic mass-forming cholangiocarcinoma; SD, standard deviation.

A relatively high DWI signal was presented in 29 patients (67.5%, 29/43) in the IMCC group (**Figures 2A–E**) and in 15 patients (60.0%, 15/25) in the atypical liver abscess group (**Figures 3A–E**), with no significant (P=0.54) differences between the two groups (**Table 2**). However, all these 29 patients (100.0%) with a relatively high DWI signal in the IMCC group presented with a relatively high peripheral signal with no one having a relatively high central signal, whereas only one (6.7%, 1/15) of the 15 patients with a relatively high DWI signal in the atypical liver abscess group exhibited a relatively high peripheral signal with the other 14 (93.3%, 14/15) showing a relatively high central signal (**Table 3** and **Figure 3C**). A significant (P<0.001) difference existed in the proportion of patients presenting with this pattern of central and peripheral signal between the two groups (**Table 3**).

**Table 2:** Relative, homogeneous and heterogeneous high DWI signal between IMCCs and atypical liver abscess.

group	relatively high signal	homogeneous or heterogeneous high signal
IMCC (n=43)	29 (67.5%)	14
atypical liver abscess (n=25)	15 (60.0%)	10
*χ* ^2^	0.383	
*P*	0.536	

IMCC, intrahepatic mass-forming cholangiocarcinoma; DWI, diffusion-weighted imaging.

**Table 3:** Relative, peripheral and central high DWI signal between IMCC and atypical liver abscess.

Group (relatively high signal)	relatively high peripheral signal	relatively high central signal
IMCC (n=29)	29 (100%)	0 (0.0%)
atypical liver abscess (n=15)	1 (6.7%)	14 (93.3%)
*χ* ^2^	—	
*P*	<0.001	

The statistical method was the Fisher exact probability method, without specific test value. IMCC, intrahepatic mass-forming cholangiocarcinoma; DWI, diffusion-weighted imaging.

In the IMCC group, 23 (53.5%, 23/43) patients presented with a homogeneous high signal, five (11.6%, 5/43) exhibited heterogeneous high signals, five (11.6%, 5/43) showed a relatively high peripheral signal, and ten (23.3%, 10/43) displayed a relatively high central signal in T2WI sequence. In the atypical liver abscess group, one (4%, 1/25) patient presented with a homogeneous high signal, eight (32%, 8/25) exhibited heterogeneous high signals, no patients showed a relatively high peripheral signal, and sixteen (64%, 16/25) displayed a relatively high central signal in T2WI sequence. A significant (P=0.000) difference existed in the proportion of patients presenting with the four signals. The IMCC group was mainly characterized by uniform high signal in T2WI sequence (53.5%), while the atypical liver abscess group was mainly characterized by relatively high central signal in T2WI sequence (64%) (**Table 4**).

**Table 4:** T2WI signal characteristics between IMCCs and atypical liver abscesses.

group	relatively high peripheral signal	relatively high central signal	homogeneous high signal	heterogeneous high signal
IMCC (n=43)	5 (11.6%)	10 (23.3%)	23 (**53.5%**)	5 (11.6%)
atypical liver abscess (n=25)	0 (0%)	16 (**64%**)	1 (4%)	8 (32%)
*χ* ^2^	—			
*P*	0.000			

The statistical method was the Fisher exact probability method, without specific test value. IMCC, intrahepatic mass-forming cholangiocarcinoma. T2WI, T2-Weighted imaging.

In the IMCC group, there were five cases (11.6%, 5/43) with a lesion of low signal and 38 cases (88.4%, 38/43) with a lesion of low signal in the peripheral region but high signal in the center on the enhanced images at the HBP (**Figure 2D**). The │CNR│ was 42.76 ± 3.93 in the peripheral region but 25.81 ± 2.89 in the center. The visibility was 3.9 ± 0.7. In the atypical liver abscess group, 21 cases (84%, 21/25) had a lesion with slightly higher peripheral signal but uneven and low central signal (**Figure 3D**), and four cases (16%, 4/25) had a lesion with low peripheral signal but uneven central signal. The │CNR│ was 21.70 ± 3.88 in the peripheral region but 21.54 ± 4.75 in the center. The visibility was 2.7 ± 0.7. A significant (P < 0.05) difference was detected in the values of peripheral │CNR│ and visibility between the two groups (**Table 5**).

**Table 5:** Comparation of IMCCs and atypical liver abscess in │CNR│.

group	peripheral│CNR│ $\left(\bar{x}\;\pm \;s\right)$	center│CNR│ $\left(\bar{x}\;\pm \;s\right)$	visibility
IMCC	42.76±3.93	25.81±2.89	3.9±0.7
atypical liver abscess	21.70±3.88	21.54±4.75	2.7±0.7
*P*	0.001	0.421	<0.001

IMCC, intrahepatic mass-forming cholangiocarcinoma; │CNR│, absolute value of the contrast noise ratio.

The diagnosis of IMCC (the positive group, n=43) and atypical liver abscesses (the negative group, n=25) was shown in **Table 6** using the approaches of DWI, hepatobiliary-stage imaging, DWI combined with the enhanced HBP imaging, surgical pathology (**Figures 2E**, **3E**), and follow-up. The sensitivity of the HBP imaging was significantly (P=0.002) higher than that of DWI. The sensitivity and accuracy of DWI combined with the HBP imaging were significantly higher (P=0.002 and P<0.001, respectively) than those of either the HBP imaging or DWI alone (**Table 7**). No significant (P>0.05) difference was found in the specificity of different approaches.

**Table 6:** Diagnostic results of different modes.

methods		Surgical pathology or follow-up results		
		positive	negative	total
DWI	positive	29	1	30
	negative	14	24	38
	Total	43	25	68
Hepatobiliary-stage imaging	positive	38	4	42
	negative	5	21	26
	Total	43	25	68
DWI and hepatobiliary- stage imaging	positive	39	0	39
	negative	4	25	29
	total	43	25	68

DWI, diffusion-weighted imaging.

**Table 7:** Comparison of diagnostic sensitivity, specificity, and accuracy between different modes (%).

methods	Sensitivity	specificity	accuracy
DWI	67.4 (29/43)	96.0 (24/25)	77.9 (53/68)
Hepatobiliary-stage imaging	88.4 (38/43)*	84.0 (21/25)	86.8 (59/68)
Combination of two	90.7 (39/43)**	100.0 (25/25)	94.1 (64/68)**

*P<0.05 between hepatobiliary-stage imaging and DWI. **P<0.01 between DWI combined with hepatobiliary-phase imaging and DWI alone and between DWI combined with hepatobiliary-phase imaging and hepatobiliary-phase imaging alone. DWI, diffusion-weighted imaging.

## Discussion

In our study, 67.5% (29/43) of the IMCC lesions presented with relatively high signals, all of which exhibited a relatively high peripheral signal (100.0%) on DWI images, whereas 60.0% (15/25) of the atypical liver abscess group demonstrated a relatively high signal, with only one displaying homogeneous high peripheral signal but 14 (93.3%, 14/15) showing a relatively high signal in the center. The lower the degree of differentiation of an IMCC lesion, the greater the probability of a target ring (20).The high signal in the center of an atypical liver abscess is caused by denatured and necrotic neutrophils and necrotic dissolved tissue debris (5, 6). Joo et al. (21) found that detection of IMCC in the DWI sequence had a probability of 68.5%, consistent with our results. However, Park et al. (20) and Min et al. (14) found that the probability of detecting the target sign of IMCC in the DWI sequence was 75% and 83.5%, respectively, much higher than that in our study. This may be caused by lower differentiation of the lesion in the cases collected in the study by Park et al, thus leading to a high probability of detecting the target sign. In addition, the DWI signal intensity is affected by T2WI signal intensity changes (T2 penetration effect and T2 darkening effect). Dense collagen or coagulative necrosis in the focal center of IMCC reduces T2WI signal intensity, which can be directly transformed into the central dark area on DWI (14). Areas containing loose fibrous tissue or necrosis can result in increased diffusivity and central dark areas with high b-value (14). The ADC (apparent diffusion coefficient) values of IMCC lesions at different b values were lower than those of atypical liver abscesses (22).

On the HBP imaging, an IMCC lesion is more likely to present with (about 88.4%, 38/43) a low peripheral signal but a high central signal (target sign), which is due to accumulation of contrast agent in the fibrous tissue. The edge of the tumor lesion lacks normal functioning liver cells and is unable to absorb the liver cell specific contrast agent (9, 14–18, 23, 24). Atypical liver abscess lesions (about 84%,21/25) may present with a peripheral slightly higher signal and a low and uneven central signal. Because the peripheral part of an atypical liver abscess is mainly composed of hepatocytes with a zone of inflammatory edema and granulation tissue, which has some normal hepatocyte function, the contrast agent can be taken up (8). The boundary between an IMCC lesion and the liver parenchyma on the HBP imaging was clear, which was more striking in the IMCC lesion than that of an atypical liver abscess. The IMCC lesion is most clearly displayed on the HBP imaging (25). The peripheral signal of an atypical liver abscess was slightly higher than that of the liver parenchyma, and the lesion seems to have signs of reduction in size. Some CT-related research had found that the atypical liver abscess lesions showed peripheral equal density at the delayed stage and seemed to decrease in size compared with those seen on plain CT imaging, which is referred to as a shrinkage sign (26–28). The shrinking sign of the liver abscess lesion reflects the inflammatory reaction of suppurative inflammation or residual liver tissue, caused by slow infiltration and clearance of contrast agent in pyogenic granulation tissue (26, 28). Based on these findings, the atypical liver abscess lesion may also have a mass shrinkage sign on the HBP imaging, with equal or slightly higher signal boundaries.

There have been few reports on the differential diagnosis between IMCCs and atypical liver abscesses using DWI combined with the HBP enhanced imaging of Gd-BOPTA. In our study, it was found that the sensitivity of the HBP imaging was significantly higher than that of DWI. The sensitivity and accuracy of DWI combined with the HBP imaging were significantly higher than those of either the HBP imaging or DWI alone. Chen et al. (22) found that the sensitivity, specificity, and accuracy of DWI in the diagnosis of intrahepatic cholangiocarcinoma were 98.15%, 100%, and 98.82%, respectively, higher than those in our study. A possible reason is that Chen et al. used DWI signal analysis of the lesion as a whole. In their study (22), DWI signals were subdivided into a relatively high peripheral signal and a relatively high central signal, which were more distinguishable, according to the signal characteristics of the lesions. Therefore, combination of DWI and HBP imaging has significantly higher sensitivity and accuracy than those of a single technique alone.

In the pathological study, IMCC lesions mainly presented with heterogeneous epithelial cells in the fibrous tissue which may demonstrate mucinous degeneration, whereas atypical liver abscesses have acute and chronic inflammatory cell infiltration but no lining epithelial cells. In the atypical liver abscess, the small vessels are dilated and congested. Interactions and crosstalk between malignant tumor cells and surrounding vessels are one of the pivotal physiological events in the expansion and dissemination of neoplastic cells (29). Antitumor immunity plays a very important role in this process (29). The imaging presentations of both IMCCs and atypical liver abscesses may reflect the constant crosstalk and interactions between the neoplastic cells and surrounding tissues as well as the process of neoplastic expansion. The changes of vascular structures and inflammatory cell infiltration may have some values in the differentiation of IMCCs from atypical hepatic abscesses on imaging presentations, and strategies combining anti-angiogenic therapy and immunotherapy have the potential to change the vascular structure and inflammatory cell infiltration in the neoplastic microenvironment, thus improving the treatment response and effects. Ultimately, the imaging presentations of IMCCs may be changed with use of these treatment strategies.

The results of this study can provide a theoretical basis for differentiating IMCC from atypical liver abscess and improve the diagnostic rate. At the same time, our study outcomes can also provide a basis for wide application of the liver specific contrast agent Gd-BOPTA. The relationship of different pathological types and different pathological grades of IMCCs with the image manifestations of MRI scanning sequences is still poorly investigated, and more data of patients are necessary for such studies. Hepatocyte specific contrast agents will be widely used in the next five years. Their characteristic imaging manifestations, such as target sign and multi-layer target sign, are of great values in the differentiation of liver neoplastic and non-neoplastic lesions. Moreover, the study of these liver lesions will be more detailed, including pathological type and grade as well as interaction between the lesion and the surrounding microenvironment.

Some limitations existed in our study, including the retrospective and one-center design, a small cohort of patients, and Chinese patients enrolled only. Moreover, the IMCCs of different pathological types and liver abscesses caused by different pathogens were not further divided and analyzed. These limitations may affect the generalization of this study. In order to achieve a better outcome, future studies should be designed and performed with a large cohort of patients, prospective and multi-center design, patients of different races being enrolled, and analyses of IMCCs of different pathological types and live abscesses caused by different pathogens.

In conclusion, IMCCs exhibit a relatively high peripheral signal on DWI, a homogeneous high signal on T2WI, and a typical “target sign”, with a clear boundary on the hepatobiliary-phase imaging. However, atypical liver abscesses display a relatively high central signal on DWI, a relatively high central signal on T2WI, a slightly high peripheral signal but an uneven low central signal, with no clear boundary on the hepatobiliary-phase imaging. Combination of DWI and hepatobiliary-phase enhanced imaging has higher sensitivity and accuracy than those of either technique in differentiating IMCCs from atypical liver abscesses.

## Data Availability Statement

The raw data supporting the conclusions of this article will be made available by the authors, without undue reservation.

## Ethics Statement

The studies involving human participants were reviewed and approved by Ethics Committee of Affiliated Hospital of Hebei University. The patients/participants provided their written informed consent to participate in this study.

## Author Contributions

Study design: L-HX, X-PY, and B-LG. Data collection: L-HX, L-YZ, YZ, XM, Z-PM, and Y-JZ. Data analysis: L-HX, X-PY, and B-LG. Supervision: Y-JZ. Writing of the original version: L-HX. Revision of the paper: B-LG. All authors contributed to the article and approved the submitted version.

## Funding

Hebei Provincial Science and Technology Project (192777131D); Hebei Provincial Health Commission (G2019041). Key Science and Technology Research Program of Hebei Province (20210789), the study of IVIM-DWI combined with Gd-BOPTA in hepatobiliary stage in the identification of Intrahepatic cholangiocarcinoma and atypical liver abscess; Baoding Science and Technology Bureau project (2041ZF176), study on the differential value of IVIM-DWI combined with Gd-BOPTA in hepatobiliary stage for intrahepatic mass-forming cholangiocarcinoma and atypical liver abscess.

## Conflict of Interest

The authors declare that the research was conducted in the absence of any commercial or financial relationships that could be construed as a potential conflict of interest.

## Publisher’s Note

All claims expressed in this article are solely those of the authors and do not necessarily represent those of their affiliated organizations, or those of the publisher, the editors and the reviewers. Any product that may be evaluated in this article, or claim that may be made by its manufacturer, is not guaranteed or endorsed by the publisher.
