# Corrigendum: DWI Combined With Hepatobiliary Phase Enhanced Imaging Can Better Differentiate Cholangiocarcinoma From Atypical Liver Abscesses

**DOI:** 10.3389/fonc.2022.967385

**Published:** 2022-07-21

**Authors:** Li-Hong Xing, Li-Yong Zhuo, Yu Zhang, Xi Ma, Ze-Peng Ma, Ying-Jia Zhao, Xiao-Ping Yin, Bu-Lang Gao

**Affiliations:** ^1^Department of CT/MRI Room, Affiliated Hospital of Hebei University, and Key Laboratory of Cancer Radiotherapy and Chemotherapy Mechanism and Regulations, Baoding, China; ^2^School of Clinical Medicine of Hebei University, Baoding, China

**Keywords:** intrahepatic mass-forming cholangiocarcinoma, atypical liver abscess, DWI, hepatobiliary phase, enhanced imaging

In the published article, there was an error regarding the affiliation(s) for Li-Hong Xing. As well as having affiliation(s) 1, they should also have School of Clinical Medicine of Hebei University, Baoding, China.

In the published article, there was an error in **Figure 2B** as published. The image of **Figure 2B** is wrong as it is mirror inverted. The corrected **Figure 2** and its caption appear below.

**Figure 2:**
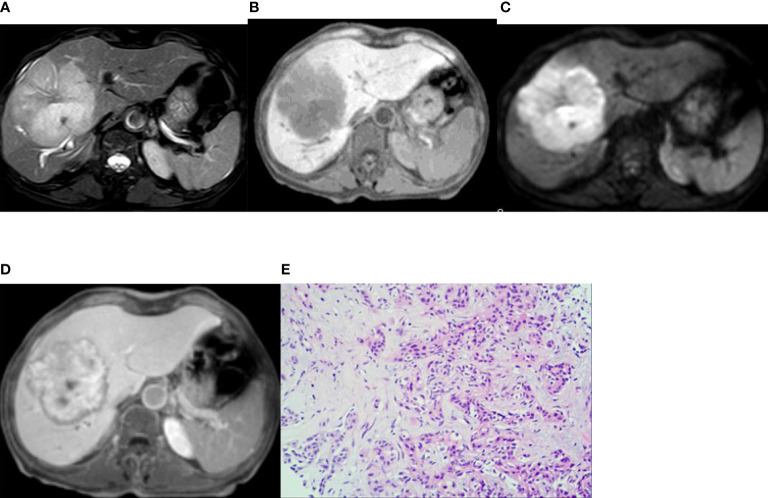
Imaging of intrahepatic mass-forming cholangiocarcinoma. A 66-year-old woman had cholangiocarcinoma with fatigue, no obvious cause of poor appetite, and laboratory examination of CA19-9 13455 U/mL. **(A, B)** (T2WI and T1WI) showed a lobulated mass with a long T1 and slightly longer T2 signal with clear boundaries. **(C)** The lesion showed a peripheral relatively high signal on DWI image. **(D)** On the hepatobiliary-phase enhanced imaging, the peripheral signal was low, whereas the central signal was high. The │CNR│ was 34.92 in the peripheral region but 20.94 in the central. The visibility score was 5. **(E)** Pathological sections showed heterogeneous epithelial cells in the fibrous tissues, some of which were glandular and in cords, with mucinous degeneration in the interstitial fibrous tissues. The pathologic diagnosis was cholangiocarcinoma (medium–poorly differentiated).

The authors apologize for these errors and state that this does not change the scientific conclusions of the article in any way. The original article has been updated.

## Publisher’s Note

All claims expressed in this article are solely those of the authors and do not necessarily represent those of their affiliated organizations, or those of the publisher, the editors and the reviewers. Any product that may be evaluated in this article, or claim that may be made by its manufacturer, is not guaranteed or endorsed by the publisher.

